# Ten simple rules for establishing a drug discovery lab in resource-limited settings

**DOI:** 10.1371/journal.pcbi.1014249

**Published:** 2026-05-07

**Authors:** Ryman Shoko, Chipampe Lombe, Tsungai Faith Manyadza, Sithulisiwe Ngwenya, Peter Mubanga Cheuka, Grace Mugumbate

**Affiliations:** 1 Department‌‌ of Basic Sciences, University of Lusaka, Lusaka, Zambia; 2 Scientific Department, Macha Research Trust, Choma, Zambia; 3 Department of Chemical Sciences, Midlands State University, Gweru, Zimbabwe; 4 Department of Pure and Applied Chemistry, University of Zambia, Lusaka, Zambia; Montreal, CANADA

## Introduction

Drug‌‌ discovery is widely recognised as one of the most resource-intensive scientific endeavours. Laboratories in resource-limited settings often face significant barriers—insufficient infrastructure, limited funding, and restricted access to technology. These constraints may appear insurmountable at first. However, necessity often drives innovation, and numerous successful initiatives across Africa and other low-resource regions demonstrate that impactful research is achievable.

Drug discovery is also a global health imperative. Neglected diseases such as schistosomiasis, leishmaniasis, and sleeping sickness continue to affect millions in sub-Saharan Africa, yet remain under-funded by global pharmaceutical pipelines [1]. At the same time, the rising incidence of noncommunicable diseases such as diabetes and cancer is placing added strain on already fragile health systems [2]. However, global investment patterns remain highly unequal. Less than 10% of global health research funding is directed towards diseases that affect 90% of the world’s population—a disparity often called the “10/90 gap” [1]. This imbalance underscores the importance of strengthening drug discovery infrastructure in low- and middle-income countries, where the disease burden is greatest, but research capacity is limited.

This article presents ten simple rules to guide the establishment of drug discovery laboratories under such conditions. Drawing from practical experience in under-resourced contexts, these principles are designed for early-career scientists, educators, and policymakers. Rather than prescriptive rules, they offer a flexible framework that supports the development of laboratories that are robust, relevant, and sustainable [3,4].

Although these rules are motivated largely by the needs of neglected and underfunded diseases, their relevance extends well beyond this domain. Many common global diseases, including cancer, metabolic disorders, and infectious diseases, show substantial regional variation in genetic, environmental, lifestyle, and pathogen-related determinants, which in turn shape disease mechanisms and therapeutic responses. Strengthening drug discovery capacity in low- and middle-income countries therefore contributes not only to addressing local health priorities but also to improving global biomedical knowledge and therapeutic strategies. Moreover, advances arising from laboratories in resource-limited settings—particularly in areas such as antimicrobial resistance, emerging infections, and pathogen diversity—have direct implications for global health security and translational research worldwide.

## Rule 1: Focus on local health needs

A clear research focus, aligned with urgent regional health priorities, is the most effective starting point. Neglected tropical diseases, antimicrobial resistance, and herbal pharmacology are especially pertinent areas in many African contexts. Addressing these health burdens increases societal relevance and strengthens funding prospects, whether from national agencies or international donors [4].

Moreover, adding health economics and implementation research builds credibility. Demonstrating cost-effectiveness and community-level impact helps cement support among funders and policymakers [4].

## Rule 2: Build a skilled and supportive team

Sustainability in drug discovery depends on people rather than equipment. Investing in capacity building through workshops, short courses, and graduate programmes lays the foundation for long-term resilience. Skills in bioinformatics, molecular biology, synthetic chemistry, pharmacology, and regulatory science are all necessary for a well-rounded team [4,5]. Building capacity also requires fostering interdisciplinary collaboration, as drug discovery sits at the intersection of biology, chemistry, medicine, and data science.

Capacity building should include training in laboratory management, quality assurance, and regulatory requirements to ensure reproducibility and compliance.

## Rule 3: Make the most of existing infrastructure

Making the most of existing infrastructure is often the most practical way to begin building drug discovery capacity. Before investing in new facilities, first assess and optimise existing institutional or regional infrastructure. Shared laboratory space, hospital collaborations, and partnerships with nearby universities can provide crucial access. These arrangements reduce costs and encourage interdisciplinary interactions, and formal agreements can ensure equipment upkeep and knowledge transfer [3].

In several African countries, genomic surveillance infrastructure established during the coronavirus disease 2019 (COVID-19) pandemic has been adapted for broader infectious disease monitoring. By 2022, more than 85% of African countries had established in-country sequencing capacity, initially developed for severe acute respiratory syndrome coronavirus 2 (SARS-CoV-2), but now applied to antimicrobial resistance and endemic diseases [6,7]. The Africa Pathogen Genomics Initiative is further strengthening regional capacity with investment in continent-wide sequencing networks [8].

## Rule 4: Form local and international partnerships

Forming both local and international partnerships is critical for accessing resources, knowledge, and mentorship. Collaborations with local universities, hospitals, and government research agencies enable resource sharing and collective impact. For example, the Holistic Drug Discovery and Development Centre (H3D) at the University of Cape Town was built through strategic partnerships linking academic groups, public funders, and product development organisations, enabling access to compound libraries, screening platforms, and translational expertise that would not have been possible within a single institution. Global partnerships with organisations like Medicines for Malaria Venture, the Drugs for Neglected Diseases initiative, and the African Academy of Sciences, as well as engagement with diaspora scientists, bring access to compound libraries, training, and mentorship [9,10].

Partnerships also enhance visibility and advocacy. Engagement with policymakers, patient groups, and communities ensures that research is responsive to societal needs. These relationships build legitimacy and can generate non-traditional support, including policy reforms and industry linkages that sustain laboratory growth.

## Rule 5: Use computational tools and open resources

Making effective use of computational tools and open resources provides a cost-effective entry point for laboratories in resource-limited settings. Open-access databases (e.g., ChEMBL, PubChem), docking tools (AutoDock, PyRx), and platforms (Galaxy, KNIME) enable virtual screening with minimal wet-lab infrastructure [4,11]. Artificial intelligence-driven tools such as AlphaFold and related machine-learning approaches can accelerate candidate identification [12–14].

Past initiatives such as H3ABioNet, a pan-African bioinformatics network for the Human Heredity and Health in Africa (H3Africa) consortium, demonstrated how collaborative bioinformatics training could equip scientists to leverage these tools effectively, leaving a legacy of capacity that continues to influence current training programmes [5,15]. Expanding access to cloud-based computing, through academic subsidies or national initiatives, lowers barriers further. Integrating traditional knowledge of medicinal plants with cheminformatics approaches enriches innovation and ensures cultural relevance [4].

## Rule 6: Start small and grow gradually

Ambition is valuable, but early over-expansion can be damaging. Many successful laboratories start with modest projects such as molecular docking studies or small biochemical screens before moving into medicinal chemistry or animal studies. These initial projects provide proof of concept, generate preliminary data, and demonstrate feasibility to funders and collaborators [4]. This staged approach is reflected in African drug discovery initiatives. For example, H3D at the University of Cape Town used malaria as an anchor programme around which broader drug discovery capacity was developed [9], while the Pan-African drug metabolism and pharmacokinetics (DMPK) Centre of Excellence at the African Institute of Biomedical Science and Technology (AiBST) in Zimbabwe has built capacity by establishing core assays and linking them to more specialised ADMET capabilities [16].

Equally important is to establish standard operating procedures (SOPs), quality control procedures, and data management systems from the outset. Even small projects benefit from these practices, which ensure rigour, reproducibility, and scalability. By embedding good laboratory practices early, teams position themselves to grow responsibly and attract further investment [3].

Incremental growth reduces risk and builds confidence. As capacity develops, laboratories can progressively expand into more resource-intensive areas such as high-throughput screening or advanced pharmacokinetics.

## Rule 7: Share knowledge and encourage mentorship

Sharing knowledge and fostering mentorship strengthens both individual researchers and the long-term resilience of the laboratory, and helps ensure that skills, standards, and institutional memory are transmitted across generations of trainees. Knowledge sharing builds both capacity and resilience. Internal seminars, data-sharing platforms, and collaborative projects ensure that expertise circulates within and beyond the laboratory. Publicly available repositories and preprint servers can also increase visibility and invite external collaboration. Lessons from the now-concluded H3ABioNet initiative underscored the importance of shared data and metadata standards, coordinated training programmes, and interoperable analysis platforms as foundations for sustainable, distributed collaboration across institutions [5,15].

Mentorship strengthens these processes further. Structured programmes that pair junior researchers with senior scientists, whether for molecular docking, assay development, or data analysis, support skill transfer and career development [17]. Recognition of contributions through co-authorship and acknowledgement motivates staff and reinforces a collaborative ethos, which is critical in resource-limited environments.

## Rule 8: Find funding from different sources

Diversifying funding is critical to resilience. Beyond conventional grants, laboratories should pursue philanthropic foundations, non-governmental organisations, and innovation hubs. The African Union, European and Developing Countries Clinical Trials Partnership, Gates Foundation, and Wellcome Trust are examples of funders that support drug discovery capacity in Africa.

Equally important is financial management. Transparent reporting, efficient budget use, and alignment of research outputs with societal priorities build credibility with funders. Innovation prizes, crowdsourcing, and public–private partnerships offer additional opportunities for resource mobilisation.

## Rule 9: Follow ethical and regulatory guidelines

Adherence to high ethical standards ensures credibility and sustainability. Engaging with institutional review boards, biosafety committees, and regulatory agencies early in the research process avoids costly delays and enhances public trust.

Ethical considerations also extend to natural products research. The Nagoya Protocol on Access and Benefit Sharing sets obligations for fair use of indigenous knowledge and genetic resources [18]. Respecting these frameworks protects communities and strengthens long-term collaborations. Building capacity in research ethics, including training institutional review boards, is a critical step for laboratories in many African countries where regulatory expertise is still developing. In addition, rigorous ethical oversight, transparent reporting, and adherence to reproducible research practices are essential to protect the scientific record, particularly in an era of increasing concern about irreproducible results, fabricated data, and misleading or non-scientific claims.

## Rule 10: Plan for sustainability and lasting impact

Sustainability should be embedded from the outset. Define metrics such as trained personnel, publications, patents, and health interventions. Long-term viability also requires exploring technology transfer, licensing agreements, and spin-off companies. For example, the H3D at the University of Cape Town has demonstrated this type of sustainability by linking academic discovery to downstream development through partnerships, technology transfer activities, and the advancement of candidate compounds into formal development pipelines [9].

Continuity planning matters deeply. Many initiatives falter when key individuals depart. Succession planning, standardised protocols, and institutional integration safeguard institutional memory. Linking laboratories to wider innovation ecosystems—including technology hubs, incubators, and biotech companies—broadens opportunities for translation and commercialisation.

Aligning research with national and regional development agendas, such as the African Union’s Agenda 2063, ensures that laboratories contribute to long-term policy priorities and attract sustained political support [19].

## Conclusion

Establishing a drug discovery laboratory in a resource-limited environment is both achievable and necessary. These ten rules provide a practical framework, but their strength lies in adaptability to local contexts. Regardless of setting, the principles of local relevance, capacity-building, partnerships, and sustainability remain central.

Such laboratories also promote scientific self-reliance, reducing dependence on external solutions and fostering local innovation capacity. Open science, through shared data, protocols, and training resources, further levels the playing field and enables researchers in low-resource settings to contribute globally. Ultimately, these rules should be adapted to each institutional and national context, ensuring that they remain responsive to local needs while also contributing to the broader global health research ecosystem.

Drug discovery in Africa and other resource-limited regions has potential not just to address regional health burdens, but to reshape the global scientific landscape. By integrating traditional knowledge with modern computational tools, these laboratories can pioneer context-sensitive models of innovation. The ultimate aim is not to replicate laboratories in high-resource settings, but to create sustainable centres of excellence that connect science directly to societal impact [4].
